# A longitudinal study of the brain structure network changes in HIV patients with ANI: combined VBM with SCN

**DOI:** 10.3389/fneur.2024.1388616

**Published:** 2024-04-17

**Authors:** Fan Xu, Juming Ma, Wei Wang, Hongjun Li

**Affiliations:** Department of Radiology, Beijing Youan Hospital, Capital Medical University, Beijing, China

**Keywords:** HIV, longitudinal study, voxel-based morphometry, structural covariance network, HIV-associated neurocognitive disorder

## Abstract

**Background:**

Despite the widespread adoption of combination antiretroviral therapy (cART) in managing HIV, the virus’s impact on the brain structure of patients remains significant. This study aims to longitudinally explore the persistent effects of HIV on brain structure, focusing on changes in gray matter volume (GMV) and structural covariance network (SCN) among patients at the Asymptomatic Neurocognitive Impairment (ANI) stage.

**Methods:**

This research involved 45 HIV patients diagnosed with ANI and 45 demographically matched healthy controls (HCs). The participants were observed over a 1.5-year period. Differences in GMV between groups were analyzed using voxel-based morphometry (VBM), while the graph theory model facilitated the establishment of topological metrics for assessing network indices. These differences were evaluated using two-sample *t*-tests and paired-sample *t*-tests, applying the network-based statistics method. Additionally, the study examined correlations between GMV and cognitive performance, as well as clinical variables.

**Results:**

Compared with HCs, HIV patients demonstrated reduced GMV in the right middle temporal gyrus and left middle frontal gyrus (FWE, *p* < 0.05), along with decreased betweenness centrality (BC) in the left anterior cingulate and paracingulate cortex. Conversely, an increase in the clustering coefficient (Cp) was observed (FDR, *p* < 0.05). During the follow-up period, a decline in GMV in the right fusiform gyrus (FWE, *p* < 0.05) and a reduction in node efficiency (Ne) in the triangular part of the inferior frontal gyrus were noted compared with baseline measurements (FDR, *p* < 0.05). The SCN of HIV patients exhibited small-world properties across most sparsity levels (Sigma >1), and area under the curve (AUC) analysis revealed no significant statistical differences between groups.

**Conclusion:**

The findings suggest that despite the administration of combination antiretroviral therapy (cART), HIV continues to exert slow and sustained damage on brain structures. However, when compared to HCs, the small-world properties of the patients’ SCNs did not significantly differ, and the clustering coefficient, indicative of the overall information-processing capacity of the brain network, was slightly elevated in HIV patients. This elevation may relate to compensatory effects of brain area functions, the impact of cART, functional reorganization, or inflammatory responses.

## Introduction

Acquired Immunodeficiency Syndrome (AIDS) is an infectious disease predominantly characterized by T cell immunodeficiency, caused by Human Immunodeficiency Virus (HIV) infections. HIV initially targets the immune system through macrophages (1), with the virus first being detected in lymph nodes, then in plasma within 5 days, and in cerebrospinal fluid after 8 days (2). These observations indicate that HIV can penetrate the blood–brain barrier early in the infection, thereby gaining access to the central nervous system. Once inside, HIV infects CD4+ T cells, macrophages, and microglia, inducing a continuous low-level inflammatory response in the neurons. This persistent inflammation can lead to chronic damage, including axonal injury and demyelination, ultimately contributing to the development of HIV-associated neurocognitive disorder (HAND) (3, 4). Recently, the prevalence of AIDS in China has declined due to the widespread adoption of combined antiretroviral therapy (cART), which has significantly reduced HIV-related opportunistic infections and tumors (5). Nonetheless, AIDS has evolved into a chronic, incurable condition, markedly affecting the life quality and prognosis of patients with HIV (6).

HAND is classified into three stages according to the Frascati criteria: asymptomatic neurocognitive impairment (ANI), mild neurocognitive disorder (MND), and HIV-associated dementia (HAD) (7). Nationally, the prevalence of HAND among HIV patients ranges from 15 to 55% (8). ANI is recognized as the most common form of cognitive impairment worldwide, accounting for 23.5% of cases, with a notably high occurrence in the Chinese population (9). Despite the advantages of cART early and maintaining it, which delays brain tissue damage, HAND continues to persist and progress. A number of patients will inevitably progress to severe cognitive impairment, drastically affecting their quality of life. The precise mechanisms underlying this progression are still not fully understood.

Magnetic resonance imaging (MRI), as a non-invasive and objective method, is invaluable for investigating structural brain changes in HIV patients and elucidating the underlying mechanisms. Voxel-based morphometry (VBM) studies have revealed significant bilateral thalamus volume atrophy during the ANI stage (10). Additionally, alterations in brain structure induced by HIV during the ANI stage are noted to first manifest in the basal ganglia region, particularly the shell nucleus and caudate nucleus (11). Structural covariance network (SCN) analysis, which focuses on gray matter structure, reflects anatomical connectivity and offers insights into the coordination between various brain regions and cortical states (12, 13). Consequently, analyzing brain gray matter anatomy to study SCN in cognitive disorders has become common in neuropsychiatric research (14, 15). Compared to normal controls, HIV patients have shown lower clustering coefficients and diminished structural separation, integration, and connectivity (16). However, the predominance of cross-sectional studies and the lack of consistency pose challenges in accurately delineating the mechanisms of early HIV-related damage to the parasympathetic system. As a result, these studies have not established a robust basis for early clinical diagnosis and treatment. Hence, our study monitored ANI-stage patients undergoing cART for an average duration of 1.5 years. We utilized both VBM and SCN analyses to achieve the following objectives: (1) to document both cross-sectional and longitudinal changes in gray matter volume (GMV) and SCN in patients; (2) to examine the association between GMV and cognitive performance as well as clinical variables; (3) to investigate the pathological mechanisms and progression of disease development, and to identify potential imaging biomarkers for early diagnosis.

## Materials and methods

### Participants

In this study, we enrolled 45 male HIV patients at the ANI stage and 45 demographically matched healthy controls (HCs). The HIV patients were prospectively observed for an average duration of approximately 1.5 years, from October 2021 to August 2023, at the outpatient clinic of the Infection Center at Beijing You’an Hospital. The study’s protocol received approval from the Ethics Committee of Beijing You’an Hospital and was conducted in alignment with the Declaration of Helsinki principles, with all participants providing signed informed consent. The inclusion criteria for the HIV patients were as follows: (1) Chinese males; (2) aged between 29 and 51 years; (3) right-handed; (4) having received stable cART for at least 6 months with an undetectable plasma viral load. The criteria for HCs included healthy individuals aged between 25 and 50 years. The exclusion criteria were designed to eliminate participants with potential confounding factors: (1) Presence of central nervous system (CNS) tumors, infections, cerebrovascular disease, or other systemic diseases; (2) history of neurological or psychiatric conditions such as anxiety or depression; (3) history of alcohol or drug abuse; (4) contraindications to MRI, including claustrophobia or the presence of mechanical valves. All participants underwent a standardized physical examination and provided comprehensive demographic information, including age, education, past medical history, current and childhood city of residence. For the patient group, biochemical examination indicators, including plasma viral load, current CD4+ T-cell count, and CD4/CD8 ratio, were collected from medical records.

### Neuropsychological tests

In this study, patients underwent comprehensive neurocognitive assessment utilizing sets of scales covering six cognitive domains as follows: (1) Speed of information processing (Trail marking test A); (2) learning and delayed recall (Hopkins verbal learning test and brief visuospatial memory test); (3) attention/working memory (Continuous performance test-identical pair, Wechsler memory scale and paced auditory serial addition test); (4) executive function (Wisconsin card sorting tests); (5) fine motor skills (Grooved pegboard); (6) verbal fluency (animal verbal fluency test). Additionally, participants completed a self-administered questionnaire on cognitive assessment of daily living activities to gauge the impact of cognitive impairments on daily living functions. The initial scores for each test were converted to T-scores and adjusted for gender, age, city of growth, and education level. An averaged T-score was calculated for each cognitive domain assessed by these tests. A diagnosis of ANI is considered if the performance was more than one standard deviation and < 2 standard deviations below the mean for a particular cognitive domain without a decline in daily functioning (7).

### Structural MRI acquisition

MRI data were acquired using a Siemens 3.0 T magnetic resonance scanner (Siemens Trio Tim B17 software, Germany) equipped with a 32-channel head coil. Initially, to exclude intracranial lesions, a T2-weighted fluid attenuation inversion recovery combined with fat saturation sequence (T2-FLAIR) was acquired with the following parameters: Repetition time (TR) = 8,000 ms, echo time (TE) = 97 ms, inversion time (TI) = 2370.9 ms. Subsequently, 3D-T1-weighted images (3D-T1W1) were acquired utilizing a magnetization-prepared rapid gradient echo (MP-RAGE) sequence with the following parameters: repetition time (TR) = 1,900 ms, echo time (TE) = 2.52 ms, inversion time (TI) = 900 ms, acquisition matrix = 256 × 246, field of view (FOV) = 250 × 250 mm, flip angle = 9°, voxel size =1 × 0.977 × 0.977 mm^3^, and number of slices = 176.

### Image preprocessing

The MRI data underwent the following preprocessing processes: (1) Initially, the original MRI images were converted to the Neuroimaging Informatics Technology Initiative (NIFTI) format to facilitate further processing. Each subject’s images underwent a meticulous review to identify and exclude any with artifacts or atypical brain structures. (2) Image preprocessing was executed using the Computational Anatomy Toolbox [version number:12.8.2(r2137) (CAT12) within the Statistical Parametric Mapping] (version number:7219) (SPM12) software, operating on Matlab R2018b platform. The images were normalized and aligned to MNI152 space utilizing the DARTEL algorithm. (3) Segmentation into gray matter, white matter, and cerebrospinal fluid was performed. An inspection of the segmented images confirmed the absence of artifacts or mislocalization. (4) Finally, the normalized gray matter images were smoothed using a Gaussian filter with a 12 × 12 × 12 mm full-width at half-maximum (FWHM) to enhance the quality of the statistical analysis.

### Construction of structural covariance network

For the construction of the SCN and the computation of topological metrics, we utilized the Brain Connectivity Toolbox (BCT) within the MATLAB environment. The brain was segmented into 90 distinct regions based on the Anatomical Automatic Labeling (AAL) template, from which the GMV of each region was extracted. These regions were then designated as network nodes. Age and total intracranial volume were incorporated as covariates in our analysis. Pearson’s correlation coefficients among the GMV of all brain regions were calculated, resulting in a 90 × 90 correlation matrix. This matrix was subsequently transformed into a binary matrix by applying a specific sparsity threshold, which facilitated the analysis of graph theoretical parameters. Following the guidelines of a previous study (17), the final sparsity threshold ranged from 0.05 to 0.50, with an interval of 0.05, ensuring the absence of isolated nodes in the fully connected network. After 1,000 substitution tests, the gray matter covariance network was compared with a random network to validate the non-randomness of the network’s topological properties, and 100 random networks were generated for this purpose. Employing a graph-theoretical framework, we calculated both global and nodal network properties across each sparsity level using the area under the curve (AUC) method. Global network characteristics included average path length (Lp), clustering coefficient (Cp), global efficiency (Eg), local efficiency (Eloc), and small-world properties. Nodal attributes assessed were degree centrality (Dc), betweenness centrality (Bc), and nodal efficiency (Ne) (18).

### Statistical analysis

#### Demographic, clinical data, and neurocognitive tests

All data underwent statistical analysis using SPSS 26.0. To evaluate the distribution of variables, the Shapiro–Wilk test was initially applied. Variables that adhered to a normal distribution were presented as the mean ± standard deviation (SD), whereas those not following a normal distribution were expressed as the median and interquartile range (IQR). For comparing differences between groups, the Mann–Whitney U test was employed for variables not normally distributed, and the two-sample *t*-test was used for variables with a normal distribution. Additionally, the paired-sample *t*-test was utilized to conduct within-group comparisons. A significance level of *p* < 0.05 was considered statistically significant.

### Gray matter volume

In the cross-sectional analysis, the two-sample t-test was used for between-group comparisons (patients vs. HCs); In the longitudinal analysis, the paired-sample t-test was applied for within-group comparisons (patients before vs. after follow-up). In both of the above analyses total intracranial volume and education were considered as covariates, and all the results were subjected to correction for multiple comparisons using family-wise error correction (FWE, *p* < 0.05).

### Structural covariance network

The paired-sample t-test and the two-sample t-test, employing network-based statistics (NBS), were implemented to evaluate the differences in the area under the curve (AUC) of all network indices between groups (19), the two-sample t-test was used for the cross-sectional analysis and the paired-sample t-test was applied for the longitudinal analysis. To correct for multiple comparisons of network metrics differences between the groups, the false discovery rate (FDR) module within NBS was utilized, incorporating 1,000 permutations. A significance threshold was established at *p* < 0.05.

### Correlation analysis of imaging indices with demographic, clinical data, and neurocognitive tests

Following the extraction of the GMV from discrepant brain regions utilizing the DPABI toolkit, either Pearson’s or Spearman’s rank correlation test was chosen to investigate the relationship between the imaging indices and demographic, clinical data, as well as neurocognitive tests, contingent upon their conformity to a normal distribution. A significance level of *p* < 0.05 was considered statistically significant.

## Results

### Demographic, clinical data, and neurocognitive tests

Our study comprised 45 HCs and 45 patients in the ANI stage. During the follow-up period, there were significant increases in CD4+ T-cell counts and CD4/CD8 ratios, alongside declines in two cognitive domains among patients, relative to baseline measurements. In contrast, HIV patients demonstrated lower scores across all cognitive assessments compared to HCs. Table 1 provides detailed demographic information, clinical data, and neurocognitive scores for all participant groups.

**Table 1:** Demographic information, clinical data, and neurocognitive scores.

	HIV patients		HCs	*p*-value	
	Baseline	Follow-up		Baseline vs. HCs	Baseline vs. Follow-up
Age(years)	39.0(32.0, 41.0)	40.5(33.5, 42.5)	37.0(33.0, 42.5)	0.86^b^	NA
Education level (years)	16.0(12.0, 16.0)	16.0(12.0, 16.0)	16.0(15.0, 16.0)	0.92^b^	NA
TND, n(%)	41(91%)	43(96%)	NA	NA	NA
CD4^+^T (cells/μL)	560.6 ± 258.9	647.4 ± 256.8	NA	NA	0.001^a^
CD4/CD8 ratio	0.6(0.5, 0.9)	0.8(0.6, 0.9)	NA	NA	<0.001^b^
Scores of cognitive performance (T score)					
Speed of information processing	47.4 ± 9.1	43.6 ± 9.7	48.0(44.0, 54.0)	0.44^c^	0.011^a^
Memory (learning/delayed recall)	42.0(36.0, 46.5)	40.6 ± 7.7	47.6 ± 5.9	0.002^b^	0.935^b^
Executive function,	43.0(36.0, 49.5)	43.0(37.0, 50.0)	47.0(40.0, 51.5)	0.017^b^	0.186^b^
Attention/working memory	40.0(35.0, 43.5)	40.6 ± 8.1	45.2 ± 6.7	<0.001^b^	0.576^b^
Fine motor	43.4 ± 9.9	43.6 ± 8.1	48.0(44.0, 50.0)	0.006^b^	0.009^a^
Verbal fluency	49.9 ± 6.8	49.4 ± 7.9	51.0(46.5, 58.0)	0.301^b^	0.687^a^

HCs, healthy controls; TND, virus undetectable; NA, not applicable. ^a^Paired-sample *t*-test; ^b^Mann–Whitney U test; ^c^two-sample *t*-test.

### Gray matter volume

**Figure 1:**
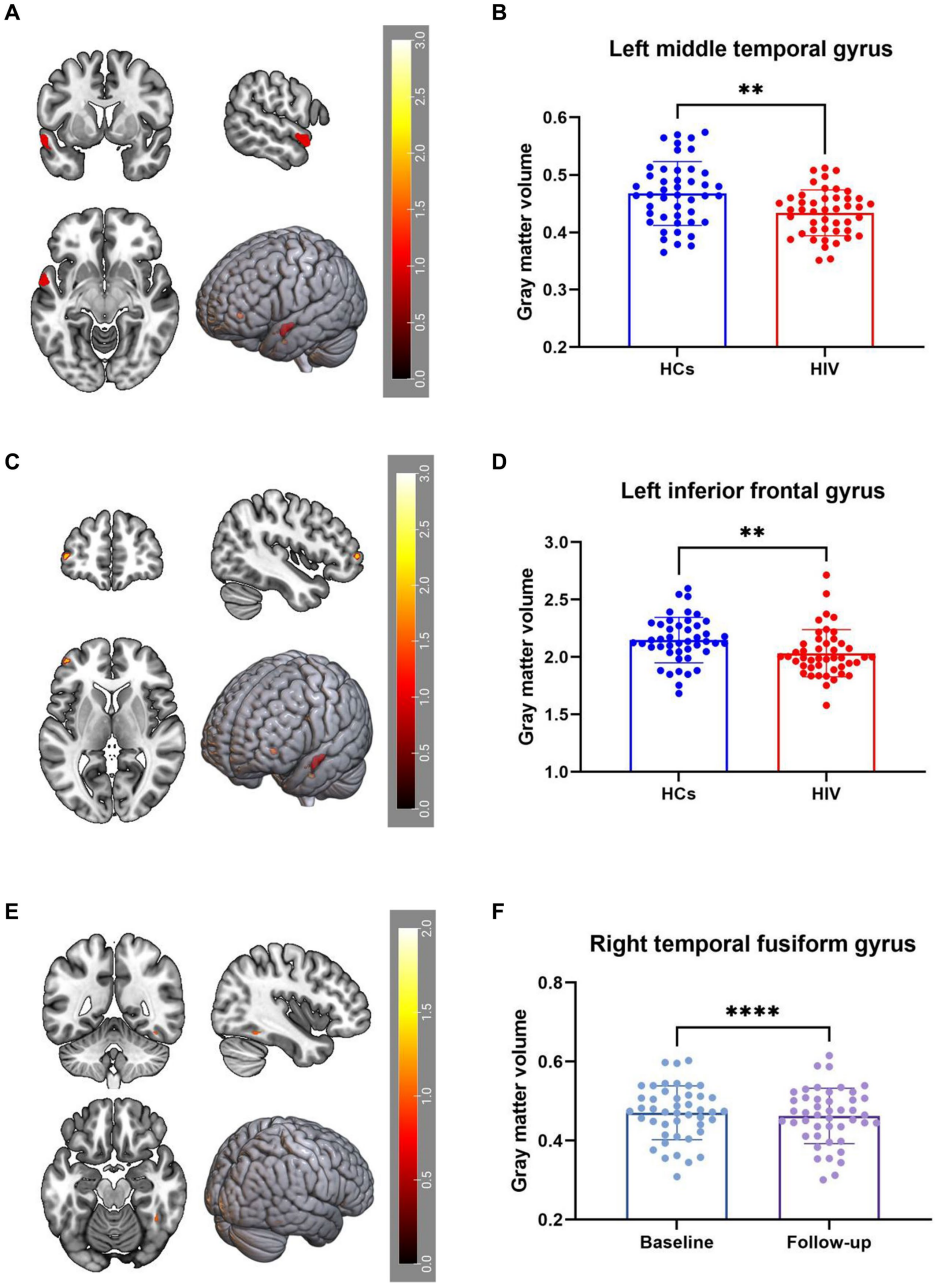
**(A–D)** Compared with HCs, HIV patients had reduced GMV in the Temporal_Mid_L and Frontal_Mid_2_L. **(E,F)** GMV in the Fusiform_R decreased during the patient’s follow-up period compared to the baseline period, all results were corrected for multiple comparisons (FWE, *p* < 0.05).

VBM analysis identified a decrease in GMV in the right middle temporal gyrus (Temporal_Mid_L) and left middle frontal gyrus (Frontal_Mid_2_L) among ANI patients compared to HCs (Figures 1A–D). Furthermore, GMV in the right fusiform gyrus (Fusiform_R) decreased during the follow-up period compared to the baseline period (Figures 1E,F). These findings were corrected for multiple comparisons (FWE, *p* < 0.05). Table 2 provides detailed differential Brain regions’ GMV information for all groups.

**Table 2:** Differences in gray matter volume between groups.

Group	Brain area	MNI coordinate (mm)			*T*-value	Cluster size
		*X*	*Y*	*Z*		
Baseline vs. follow-up	Fusiform_R	40.5	−46.5	−16.5	5.96	18
HIV vs. HCs	Temporal_Mid_L	−61.5	3	−10.5	5.41	243
	Frontal_Mid_2_L	−46.5	51	3	5.15	32

(X,Y,Z) represents the MNI coordinate location where the peak signal intensity of the cluster is located.

### Structural covariance network

**Figure 2:**
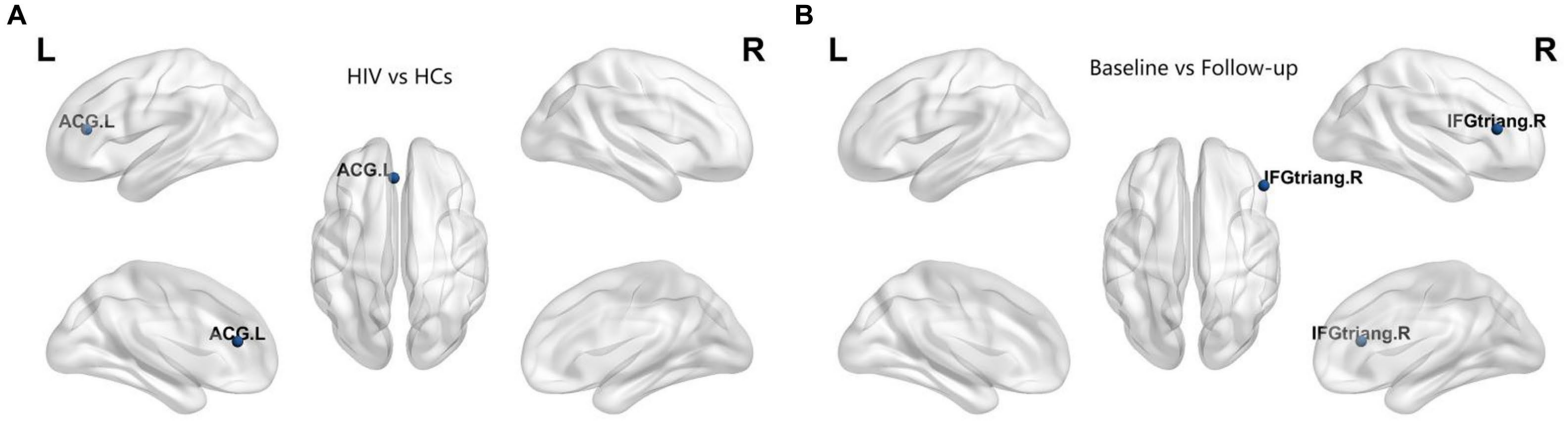
**(A)** Compared with HCs, HIV patients had decreased betweenness centrality (BC) in the ACG-L. **(B)** Compared with baseline period, HIV patients had decreased node efficiency (Ne) in the IFGtriang-R during the follow-up period (FDR, *p* < 0.05). ACG-L, anterior cingulate and paracingulate cortex; IFGtriang-R, right triangular part of the inferior frontal gyrus.

**Figure 3:**
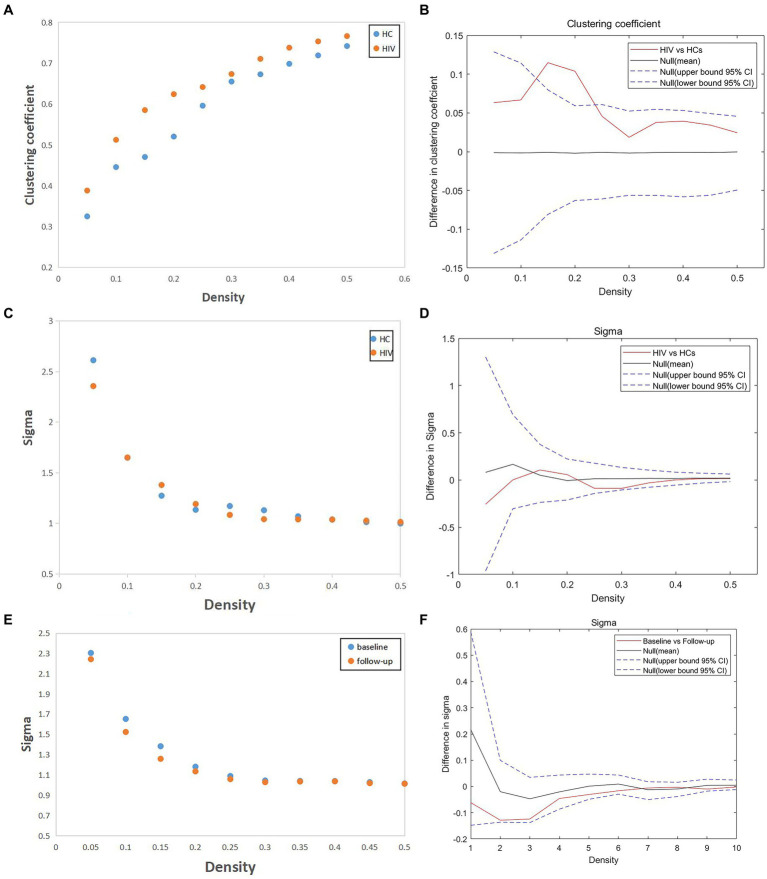
**(A,B)** Compared with HCs, HIV patients had increased clustering coefficient (Cp) over a sparsity range of 0.05–0.5, and the difference was statistically significant over a range of 0.1–0.2 (FDR, *p* < 0.05). **(C–F)** The SCN of HIV patients have the small-world characteristic at most sparsity, and AUC analysis showed no statistical difference between groups (both patients vs. HCs and patients before vs. after follow-up) (FDR, *p* > 0.05).

In our study, we assessed differences in global and nodal network properties, alongside small-world characteristics, across a range of sparsity values (0.05:0.05:0.5) within the SCN. We also examined the differences in the area under the curve (AUC) values for global and nodal network characteristics and small-world properties across this sparsity range. Compared with HCs, HIV patients demonstrated a reduction in betweenness centrality (BC) in the anterior cingulate and paracingulate cortex (ACG-L) (Figure 2A), while clustering coefficients (Cp) were increased over a sparsity range of 0.05–0.5, and the difference was statistically significant over a range of 0.1–0.2 (Figures 3A,B) (FDR, *p* < 0.05). During the follow-up period, a decrease in nodal efficiency (Ne) was observed in the right triangular part of the inferior frontal gyrus (IFGtriang-R) for HIV patients compared to the baseline measurements (Figure 2B) (FDR, *p* < 0.05). The SCN of HIV patients maintained a small-world characteristic across sparsity levels, with Sigma >1, and AUC analysis revealed no statistical difference between groups (Figures 3C–F) (FDR, *p* > 0.05). Those negative results for brain network metrics not corrected by multiple comparisons will be placed in Supplementary material.

### Correlation analysis

**Figure 4:**
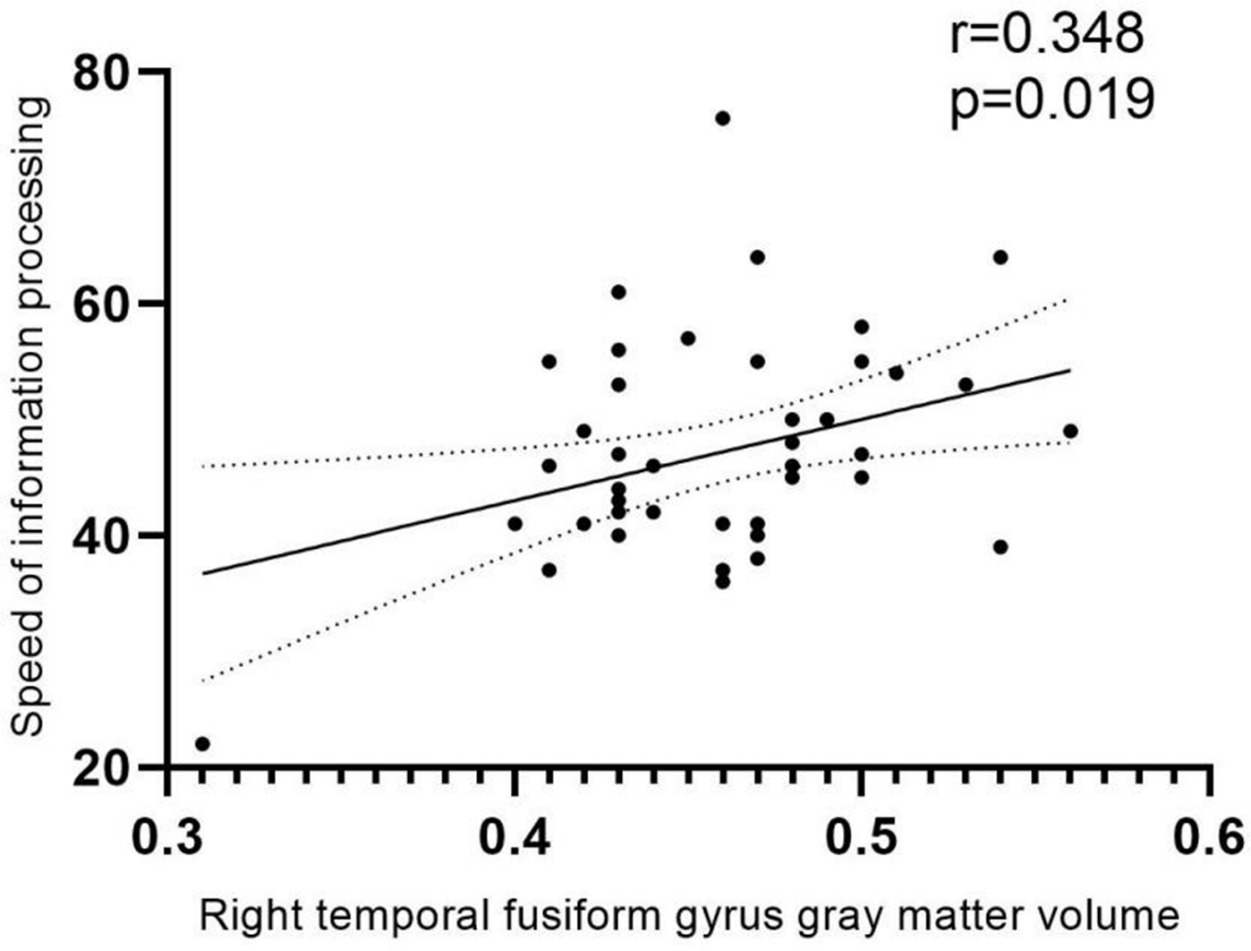
Correlation analysis. Positive correlation between GMV and speed of information processing in patients during the follow-up period (*r* = 0.348, *p* = 0.018). The area between the two dashed lines represents the 95% confidence interval.

GMV in the differential brain regions was correlated with clinical data and scores on each of the six neurocognitive tests (Figure 4). The result demonstrated a positive correlation between GMV in the Fusiform-R and the speed of information processing in patients during the follow-up period (*r* = 0.348, *p* = 0.018).

## Discussion

This study investigates the differences in voxel-based morphometry (VBM) and structural covariance network (SCN) parameters between HIV patients and healthy controls (HCs), as well as within the HIV patient group across baseline and follow-up assessments. The results uncover significant changes in cognitive functions and various structural metrics within the patient group.

### VBM results analysis

Existing literature consistently highlights impairments in both subcortical and cortical structures among HIV-infected individuals (10, 20). In our study, both cross-sectional and longitudinal, patients exhibited a reduction in GMV in the temporal lobe, aligning with the findings of Yu et al. (21) Their research demonstrated that HIV patients undergo cortical thinning in the right temporal lobe during adolescence, with longitudinal studies revealing reductions in GMV and cortical thinning primarily in the frontal and parietal lobes. Notably, Ruiz-Saez et al. reported significant thinning in the fusiform gyrus in young adults with perinatally acquired HIV (PHIV) when compared to HCs (22). Microglia, vital components of the central nervous system, may alter cortical gray matter structure via abnormal synaptic pruning, neuron phagocytosis under stress, and diminished secretion of neurotrophic factors (23, 24), potentially explaining the GMV reduction in the temporal lobe noted in our study. The temporal pole, implicated in the initial phases of neurodegenerative diseases (25), such as amnestic mild cognitive disorders and Alzheimer’s disease, plays a role in executive functions including working memory (26) and is associated with impulsivity and depression (27). Our correlation analysis revealed that a reduction in GMV in the right fusiform gyrus of patients positively correlates with a diminished speed of information processing, underscoring the influence of structural damage to gray matter on cognitive functions. A meta-analysis of patients with asymptomatic carotid stenosis found that (28), the unbalanced hemispheric atrophy in the posterior middle temporal cortex is crucial in mediating relationship between WMH burden and verbal recall memories. In addition, there have been some studies (29, 30) have demonstrated that the posterior middle temporal cortex plays a crucial role in semantic retrieval, verbal memory, and multiple advanced network systems. All of these studies and our study have shown that the temporal lobe does play a large role in cognitive function, as for the connection between different gyrus of the temporal lobe and specific cognitive functions, further research is needed.

Despite adherence to cART during the follow-up period, patients experienced mild cortical GMV reduction, which could be attributed to two primary factors. Firstly, the limited ability of cART to penetrate the blood–brain barrier facilitates the continued presence of HIV in the cerebrospinal fluid, causing persistent neuroinflammation and neuroglial activation, that result in sustained brain damage (31, 32). Secondly, some cART regimens are associated with cognitive impairment, potentially altering brain function by affecting immune cells, glial cells, or endothelial cells (33). A randomized controlled study (34) indicated that subjects on efavirenz (EFV)-containing regimens experienced lesser improvements in the speed of information processing and executive functioning compared to those not on EFV.

Additionally, it is essential to acknowledge that a study has shown the reduction in gray matter volume (GMV) in the frontal and temporal cortex of multiple sclerosis patients could be partly due to white matter damage. The interplay between the development of white matter axons and myelin sheaths is critical for the synchronized development of gray matter (35). Recent findings also indicate that disruptions in the structural connectivity of white matter, particularly among the frontal, temporal, and occipital lobe regions, underlie the pathogenesis of HIV-related brain damage. This suggests that white matter lesions may play a role in the abnormal gray matter morphology observed in HIV patients (36). However, further investigation is needed to elucidate the intricate relationship between white matter integrity and gray matter volume.

### SCN results analysis

If a node’s nearest neighbors are also directly connected to each other, they form a cluster, and the clustering coefficient quantifies the number of connections that exist between the nearest neighbors of a node as a proportion of the maximum possible number of connections (37). Thus the clustering coefficient becomes an important parameter to measure whether there is a strong local integration function in the network, highlighting the capability for local information processing within tightly interconnected brain regions (38). As mentioned in the results, the Cp values of HIV were higher than those of HCs in all sparsities, but this difference was only statistically significant in some of the sparsity intervals, which suggests that the Cp values of patients with HIV, although elevated, were not elevated to a significant extent compared to HCs, which indicating mildly enhanced functional separation local specialization, and modular processing in the SCN of HIV patients. This finding diverges from previous research that reported a diminished capacity for functional segregation in the brain networks of HIV patients. However, an insightly magnetoencephalography could explain our results (39). It indicated that HIV patients exhibited reduced θ activity in the postcentral gyrus but increased α activity in the prefrontal cortex compared to HCs. This suggests that, despite HIV-related damage, the brain is functioning more cohesively as a network rather than operating individually. Some studies propose that the increased ability for functional segregation (elevated Cp) might act as a compensatory mechanism in response to brain network damage. Various factors, such as neurotrophic factors released by local immune cells or the effects of cART, may stimulate neuronal regeneration, although the exact mechanism remains unclear.

In the limbic system, Bc decreased in the anterior cingulate cortex (ACG-L) of HIV patients, indicating less efficient information transfer among network nodes due to diminished integration. Our findings suggest that the topology of brain regions of the limbic system in HIV patients has been compromised. Furthermore, HIV patients exhibited reduced GMV in the left middle frontal gyrus (Frontal_Mid_2_L) compared to HCs and decreased Ne in the right triangular inferior frontal gyrus (IFGtriang-R) during the follow-up period. Fields et al. (40) identified a reduction in mitochondrial fission protein within the frontal cortex tissues of donors with HAND. This reduction was accompanied by the presence of enlarged and elongated mitochondria localized to the soma of damaged neurons. Under normal conditions, the distribution and elongation of mitochondria are typically balanced. Consequently, an imbalance in mitochondrial dynamics, specifically in fission and fusion processes, may lead to dysfunction and eventual cell death (41). These findings propose that an imbalance in mitochondrial fission/fusion dynamics could serve as a compensatory mechanism contributing to neurodegeneration in HIV patients. The frontal cortex, richly supplied by monoaminergic, cholinergic, and histaminergic pathways and housing a diverse population of modulatory interneurons, also projects extensively to other cortical and subcortical areas (42, 43), making it a pivotal hub for regulating essential functions such as mood, cognition, and social behavior. This could potentially explain why HIV patients exhibit dysfunction across several cognitive domains relative to HCs in our study.

The brain exhibits small-world characteristics, characterized by a shorter path length than a regular network, along with higher clustering coefficients compared to a random network. This unique arrangement facilitates both modular and integrated information processing, maximizing efficiency in information transfer at a relatively low cost. In our study, the SCN of the HIV patients in the ANI stage exhibited small-world properties similar to those of HCs, with no significant differences observed either at baseline or during the follow-up period. This suggests that, despite the presence of ANI, patients maintained the ability to process and transmit brain information with relatively normal efficiency. This efficiency likely stems from neuronal remodeling and the compensatory effects of brain area functions (44). This finding aligns with the results of Yadav et al.’s study on brain networks among pediatric HIV patients (45).

Examining both node and global characteristics, we noted that while some node characteristics were less efficient, the Cp was increased in HIV patients compared to HCs. Concurrently, no significant differences were observed in small-world characteristics or in several neurocognitive test scores. This suggests the possibility of neuronal remodeling occurring in the context of ongoing inflammation and gliocyte proliferation. A five-year follow-up analysis on the brain function of patients lent support to this hypothesis (46), revealing neuronal remodeling in specific pathways related to attention/working memory, delayed recall, and language recognition, aligning with our current findings. This could be closely tied to functional reorganization induced by the brain injury. Given the relatively young age of our study participants and early stage of ANI, significant changes may not have yet occurred. A long-term, longitudinal study is recommended to provide more comprehensive insights into structural brain indices over time.

This study has several limitations that should be considered. (1) Firstly, The current research is a short-term longitudinal study with a limited participant cohort, suggesting that more robust and reliable conclusions could be obtained from an extended longitudinal study featuring a larger population and multiple assessment points. (2) Secondly, lack of follow-up data for HCs prevented the exclusion of age effects on experimental results. Nonetheless, considering the brief duration of the follow-up and the relatively young age of the participants, it is posited that the effect of age on the structural changes in GMV among patients is probably less pronounced than that induced by HIV. (3) Thirdly, since the SCN was constructed at the group level in this study, exploring the correlation between the SCN’s topological parameters and individual cognitive scale scores, as well as biochemical examination results, was not possible.

## Conclusion

Despite the early and consistent administration of cART, HIV patients with ANI continue to exhibit reductions in GMV in the frontal and temporal lobes, alongside diminished nodal characteristics. These findings suggest that, even with cART, HIV continues to cause slow and sustained damage to brain structures. However, the patients’ small-world characteristics remained comparable to those of HCs, with no significant alterations detected between baseline and follow-up assessments. This stability may reflect neuronal remodeling and the compensatory mechanisms of brain functions. Notably, The increased Cp relative to HCs suggests that the overall information-processing capacity of the brain network has not decreased, with certain functions possibly enhanced due to cART, functional reorganization, or inflammatory response.

## Data availability statement

The original contributions presented in the study are included in the article/Supplementary material, further inquiries can be directed to the corresponding author.

## Ethics statement

The studies involving humans were approved by the Ethics Committee of Beijing You’an Hospital. The studies were conducted in accordance with the local legislation and institutional requirements. The participants provided their written informed consent to participate in this study.

## Author contributions

FX: Data curation, Formal analysis, Methodology, Software, Supervision, Visualization, Writing – original draft, Writing – review & editing, Investigation, Validation. JM: Methodology, Software, Writing – original draft, Investigation. WW: Supervision, Writing – original draft, Methodology, Validation. HL: Conceptualization, Data curation, Funding acquisition, Supervision, Validation, Writing – original draft, Writing – review & editing.

## References

[1] Heaton RK Marcotte TD Mindt MR Sadek J Moore DJ Bentley H. The impact of HIV-associated neuropsychological impairment on everyday functioning. J Int Neuropsychol Soc. (2004) 10:317–31. doi: 10.1017/S1355617704102130, PMID: 15147590

[2] Valcour V Chalermchai T Sailasuta N Marovich M Lerdlum S Suttichom D. Central nervous system viral invasion and inflammation during acute HIV infection. J Infect Dis. (2012) 206:275–82. doi: 10.1093/infdis/jis326, PMID: 22551810 PMC3490695

[3] Anzinger JJ Butterfield TR Angelovich TA Crowe SM Palmer CS. Monocytes as regulators of inflammation and HIV-related comorbidities during cart. J Immunol Res. (2014) 2014:569819:1–11. doi: 10.1155/2014/569819 25025081 PMC4082935

[4] Deeks SG Tracy R Douek DC. Systemic effects of inflammation on health during chronic HIV infection. Immunity. (2013) 39:633–45. doi: 10.1016/j.immuni.2013.10.001, PMID: 24138880 PMC4012895

[5] Wang Y Liu M Lu Q Farrell M Lappin JM Shi J. Global prevalence and burden of HIV-associated neurocognitive disorder: a meta-analysis. Neurology. (2020) 95:e2610–21. doi: 10.1212/WNL.0000000000010752, PMID: 32887786

[6] Cysique LA Jugé L Gates T Tobia M Moffat K Brew BJ. Covertly active and progressing neurochemical abnormalities in suppressed HIV infection. Neurol Neuroimmunol Neuroinflamm. (2018) 5:e430. doi: 10.1212/NXI.0000000000000430, PMID: 29312999 PMC5754644

[7] Antinori A Arendt G Becker JT Brew BJ Byrd DA Cherner M. Updated research nosology for Hiv-associated neurocognitive disorders. Neurology. (2007) 69:1789–99. doi: 10.1212/01.WNL.0000287431.88658.8b, PMID: 17914061 PMC4472366

[8] Saylor D Dickens AM Sacktor N Haughey N Slusher B Pletnikov M. HIV-associated neurocognitive disorder — pathogenesis and prospects for treatment. Nat Rev Neurol. (2016) 12:234–48. doi: 10.1038/nrneurol.2016.27, PMID: 26965674 PMC4937456

[9] Zhang Y Qiao L Ding W Wei F Zhao Q Wang X. An initial screening for Hiv-associated neurocognitive disorders of Hiv-1 infected patients in China. J Neurovirol. (2012) 18:120–6. doi: 10.1007/s13365-012-0089-y, PMID: 22411002 PMC3859527

[10] Li R Qi Y Shi L Wang W Zhang A Luo Y. Brain volumetric alterations in preclinical Hiv-associated neurocognitive disorder using automatic brain quantification and segmentation tool. Front Neurosci. (2021) 15:713760. doi: 10.3389/fnins.2021.713760, PMID: 34456678 PMC8385127

[11] Wright PW Pyakurel A Vaida FF Price RW Lee E Peterson J. Putamen volume and its clinical and neurological correlates in primary Hiv infection. AIDS. (2016) 30:1789–94. doi: 10.1097/QAD.0000000000001103, PMID: 27045376 PMC4925211

[12] Alexander-Bloch A Giedd JN Bullmore E. Imaging structural co-variance between human brain regions. Nat Rev Neurosci. (2013) 14:322–36. doi: 10.1038/nrn3465, PMID: 23531697 PMC4043276

[13] Raznahan A Lerch JP Lee N Greenstein D Wallace GL Stockman M. Patterns of coordinated anatomical change in human cortical development: a longitudinal neuroimaging study of maturational coupling. Neuron. (2011) 72:873–84. doi: 10.1016/j.neuron.2011.09.028, PMID: 22153381 PMC4870892

[14] Hosseini SM Mazaika P Mauras N Buckingham B Weinzimer SA Tsalikian E. Altered integration of structural covariance networks in young children with type 1 diabetes. Hum Brain Mapp. (2016) 37:4034–46. doi: 10.1002/hbm.23293, PMID: 27339089 PMC5053865

[15] Bruno JL Hosseini SMH Saggar M Quintin EM Raman MM Reiss AL. Altered brain network segregation in fragile X syndrome revealed by structural connectomics. Cereb Cortex. (2017) 27:2249–59. doi: 10.1093/cercor/bhw055, PMID: 27009247 PMC5963822

[16] Li J Gao L Wen Z Zhang J Wang P Tu N. Structural covariance of gray matter volume in Hiv vertically infected adolescents. Sci Rep. (2018) 8:1182. doi: 10.1038/s41598-018-19290-5, PMID: 29352127 PMC5775353

[17] Zhang J Wang J Wu Q Kuang W Huang X He Y. Disrupted brain connectivity networks in drug-naive, first-episode major depressive disorder. Biol Psychiatry. (2011) 70:334–42. doi: 10.1016/j.biopsych.2011.05.018, PMID: 21791259

[18] Wang JH Zuo XN Gohel S Milham MP Biswal BB He Y. Graph theoretical analysis of functional brain networks: test-retest evaluation on short- and long-term resting-state functional Mri data. PLoS One. (2011) 6:e21976. doi: 10.1371/journal.pone.0021976, PMID: 21818285 PMC3139595

[19] Zalesky A Fornito A Bullmore ET. Network-based statistic: identifying differences in brain networks. NeuroImage. (2010) 53:1197–207. doi: 10.1016/j.neuroimage.2010.06.041 20600983

[20] Ragin AB du H Ochs R Wu Y Sammet CL Shoukry A. Structural brain alterations can be detected early in Hiv infection. Neurology. (2012) 79:2328–34. doi: 10.1212/WNL.0b013e318278b5b4, PMID: 23197750 PMC3578377

[21] Yu X Gao L Wang H Yin Z Fang J Chen J. Neuroanatomical changes underlying vertical HIV infection in adolescents. Front Immunol. (2019) 10:814. doi: 10.3389/fimmu.2019.00814, PMID: 31110499 PMC6499204

[22] Ruiz-Saez B García MM de Aragon AM Gil-Correa M Melero H Malpica NA. Effects of perinatal HIV-infection on the cortical thickness and subcortical gray matter volumes in young adulthood. Medicine (Baltimore). (2021) 100:e25403. doi: 10.1097/MD.0000000000025403, PMID: 33847637 PMC8051971

[23] Hammond JW Bellizzi MJ Ware C Qiu WQ Saminathan P Li H. Complement-dependent synapse loss and microgliosis in a mouse model of multiple sclerosis. Brain Behav Immun. (2020) 87:739–50. doi: 10.1016/j.bbi.2020.03.004, PMID: 32151684 PMC8698220

[24] Rački V Petrić D Kučić N Gržeta N Jurdana K Rončević-Gržeta I. Cortical gray matter loss in schizophrenia: could microglia be the culprit? Med Hypotheses. (2016) 88:18–21. doi: 10.1016/j.mehy.2015.12.021, PMID: 26880628

[25] Herlin B Navarro V Dupont S. The temporal pole: from anatomy to function-a literature appraisal. J Chem Neuroanat. (2021) 113:101925. doi: 10.1016/j.jchemneu.2021.101925, PMID: 33582250

[26] Owens MM Duda B Sweet LH MacKillop J. Distinct functional and structural neural underpinnings of working memory. Neuroimage. (2018) 174:463–71. doi: 10.1016/j.neuroimage.2018.03.022, PMID: 29551458 PMC6908808

[27] Couvy-Duchesne B Strike LT de Zubicaray GI McMahon K Thompson PM Hickie IB. Lingual gyrus surface area is associated with anxiety-depression severity in young adults: a genetic clustering approach. eNeuro. (2018) 5:ENEURO.0153-17.2017. doi: 10.1523/ENEURO.0153-17.2017, PMID: 29354681 PMC5773884

[28] Gao L Xiao Y Xu H. Gray matter asymmetry in asymptomatic carotid stenosis. Hum Brain Mapp. (2021) 42:5665–76. doi: 10.1002/hbm.25645, PMID: 34498785 PMC8559457

[29] Xu J Lyu H Li T Xu Z Fu X Jia F. Delineating functional segregations of the human middle temporal gyrus with resting-state functional connectivity and coactivation patterns. Hum Brain Mapp. (2019) 40:5159–71. doi: 10.1002/hbm.24763, PMID: 31423713 PMC6865466

[30] Davey J Thompson HE Hallam G Karapanagiotidis T Murphy C de Caso I. Exploring the role of the posterior middle temporal gyrus in semantic cognition: integration of anterior temporal lobe with executive processes. Neuroimage. (2016) 137:165–77. doi: 10.1016/j.neuroimage.2016.05.051, PMID: 27236083 PMC4927261

[31] Whitehead N Potterton J Coovadia A. The neurodevelopment of HIV-infected infants on HAART compared to HIV-exposed but uninfected infants. AIDS Care. (2014) 26:497–504. doi: 10.1080/09540121.2013.841828 24125015

[32] Ferguson D Clarke S Berry N Almond N. Attenuated SIV causes persisting neuroinflammation in the absence of a chronic viral load and neurotoxic antiretroviral therapy. AIDS. (2016) 30:2439–48. doi: 10.1097/QAD.0000000000001178, PMID: 27258396 PMC5051525

[33] Nightingale S Winston A Letendre S Michael BD McArthur JC Khoo S. Controversies in HIV-associated neurocognitive disorders. Lancet Neurol. (2014) 13:1139–51. doi: 10.1016/S1474-4422(14)70137-1, PMID: 25316020 PMC4313542

[34] Winston A Duncombe C Li PC Gill JM Kerr SJ Puls R. Does choice of combination antiretroviral therapy (CART) alter changes in cerebral function testing after 48 weeks in treatment-naive, HIV-1-infected individuals commencing cart? A randomized, controlled study. Clin Infect Dis. (2010) 50:920–9. doi: 10.1086/650743, PMID: 20146627

[35] Han XM Tian HJ Han Z Zhang C Liu Y Gu JB. Correlation between white matter damage and gray matter lesions in multiple sclerosis patients. Neural Regen Res. (2017) 12:787–94. doi: 10.4103/1673-5374.206650, PMID: 28616036 PMC5461617

[36] for the CHARTER Group Fennema-Notestine C Ellis RJ Archibald SL Jernigan TL Letendre SL. Increases in brain white matter abnormalities and subcortical gray matter are linked to CD4 recovery in HIV infection. J Neurovirol. (2013) 19:393–401. doi: 10.1007/s13365-013-0185-7, PMID: 23838849 PMC3776609

[37] Bullmore E Sporns O. Complex brain networks: graph theoretical analysis of structural and functional systems. Nat Rev Neurosci. (2009) 10:186–98. doi: 10.1038/nrn2575 19190637

[38] Rubinov M Sporns O. Complex network measures of brain connectivity: uses and interpretations. Neuroimage. (2010) 52:1059–69. doi: 10.1016/j.neuroimage.2009.10.003, PMID: 19819337

[39] Wilson TW Heinrichs-Graham E Becker KM Aloi J Robertson KR Sandkovsky U. Multimodal neuroimaging evidence of alterations in cortical structure and function in HIV-infected older adults. Hum Brain Mapp. (2015) 36:897–910. doi: 10.1002/hbm.22674, PMID: 25376125 PMC4491915

[40] Fields JA Serger E Campos S Divakaruni AS Kim C Smith K. HIV alters neuronal mitochondrial fission/fusion in the brain during HIV-associated neurocognitive disorders. Neurobiol Dis. (2016) 86:154–69. doi: 10.1016/j.nbd.2015.11.015, PMID: 26611103 PMC4713337

[41] Scorrano L. Keeping mitochondria in shape: a matter of life and death. Eur J Clin Investig. (2013) 43:886–93. doi: 10.1111/eci.12135 23869410

[42] Millan MJ Rivet JM Gobert A. The frontal cortex as a network hub controlling mood and cognition: probing its neurochemical substrates for improved therapy of psychiatric and neurological disorders. J Psychopharmacol. (2016) 30:1099–128. doi: 10.1177/0269881116672342 27756833

[43] Lewis DA. Cortical circuit dysfunction and cognitive deficits in schizophrenia – implications for preemptive interventions. Eur J Neurosci. (2012) 35:1871–8. doi: 10.1111/j.1460-9568.2012.08156.x, PMID: 22708598 PMC3383640

[44] Qu H Wang Y Yan T Zhou J Lu W Qiu J. Data-driven Parcellation approaches based on functional connectivity of visual cortices in primary open-angle Glaucoma. Invest Ophthalmol Vis Sci. (2020) 61:33. doi: 10.1167/iovs.61.8.33, PMID: 32716501 PMC7425746

[45] Yadav SK Gupta RK Garg RK Venkatesh V Gupta PK Singh AK. Altered structural brain changes and neurocognitive performance in pediatric HIV. Neuroimage Clin. (2017) 14:316–22. doi: 10.1016/j.nicl.2017.01.032, PMID: 28224079 PMC5304232

[46] Boban J Thurnher MM Brkic S Lendak D Bugarski Ignjatovic V Todorovic A. Neurometabolic remodeling in chronic HIV infection: a five-year follow-up multi-voxel MRS study. Sci Rep. (2019) 9:19799. doi: 10.1038/s41598-019-56330-0, PMID: 31875001 PMC6930328

